# Relations between antinuclear, antineutrophil cytoplasmic, anticardiolipin antibodies and degree of neurological deficit, cerebrovascular stenosis in cerebral infarction patients

**DOI:** 10.12669/pjms.39.3.6765

**Published:** 2023

**Authors:** Huihong Meng, Xiaoming Xing, Chao Zhang, Jing Zhao, Cuijian Ren, Yonggang Liu

**Affiliations:** 1 Huihong Meng, Department of Neurology, Baoding No.1 Central Hospital, Baoding 071000, Hebei, China; 2 Xiaoming Xing, Department of Neurology, Baoding No.1 Central Hospital, Baoding 071000, Hebei, China; 3 Chao Zhang, Department of Neurology, Baoding No.1 Central Hospital, Baoding 071000, Hebei, China; 4 Jing Zhao, Department of Neurology, Baoding No.1 Central Hospital, Baoding 071000, Hebei, China; 5 Cuijian Ren, Department of Neurology, Baoding No.1 Central Hospital, Baoding 071000, Hebei, China; 6 Yonggang Liu, Department of Neurology, Baoding No.1 Central Hospital, Baoding 071000, Hebei, China

**Keywords:** Antinuclear Antibody, Antineutrophil Cytoplasmic Antibody, Anticardiolipin Antibody, Degree of Neurological Deficit, Cerebrovascular Stenosis

## Abstract

**Objective:**

To investigate the correlation of antinuclear antibody (ANA), antineutrophil cytoplasmic antibody (ANCA) and anticardiolipin antibody (ACA) with the degree of the neurological defect and cerebrovascular stenosis in patients with cerebral infarction.

**Methods:**

Clinical data of 99 patients with acute cerebral infarction (ACI) admitted to the Department of Neurology of Baoding First Central Hospital from June 2020 to December 2021 were retrospectively analyzed, and their ANA, ACA, ANCA, neurological deficit (NIHSS) scores as well as cerebrovascular stenosis were detected and assessed. Moreover, the correlation between the positive expression rates of ANA, ANCA, ACA and the degree of the neurological deficit, as well as the location and degree of cerebrovascular stenosis, were analyzed.

**Results:**

All patients had ANA, ACA, ANCA antibodies with positive rates of 68.69%, 70.71%, 69.70%, and mild, moderate, and severe cerebrovascular stenosis with incidence rates of 28.28%, 32.32%, and 39.39% respectively; Moreover, their incidence of mild, moderate, and severe neurological deficits were 15.15%, 44.44%, and 40.40%, respectively. Statistically significant differences could be observed in the degree of cerebrovascular stenosis and neurological deficit between the ANA, ACA and ANCA antibody positive group and the negative group (*p<*0.05). ANA, ACA, ANCA antibody positive was moderately positively correlated with cerebrovascular stenosis rate and NIHSS score (0.40<*r*<0.60, *p<*0.05).

**Conclusions:**

The positive rates of ANA, ACA and ANCA antibodies were higher in patients with ACI, which was closely correlated with the degree of cerebrovascular stenosis and neurological deficit.

## INTRODUCTION

Antinuclear antibody (ANA) in body cells refers to an autoantibody produced by a vital component of the nucleus and related genetic molecules. Its antibody properties vary according to different substances produced, but it mainly acts on the body’s immune system.^1^ In patients with acute cerebral infarct (ACI), ANA may affect the normal structure or function of cerebral vascular endothelial cells, giving rise to an increased risk of related diseases. ANCA and ACA are also closely related to the severity of patients with ACI. Specifically, ACA is activated after abnormal lesions in the body’s vascular structure, which promotes platelet aggregation and causes thrombosis, while ANCA may be one of the important substances that cause damage to the body’s vascular endothelial cells.^2^,^3^

In the field of clinical research on ACI, there is still room for a further in-depth discussion on the influence of relevant substances regulating the body’s own immune system. In view of this, the physiological association between ANA, ACA, ANCA antibodies and ACI should be analyzed, so that a new direction of medical guidance can be provided to improve the treatment regimen for ACI.^4^ In this study, 99 patients with ACI in the Baoding First Central Hospital were selected to investigate the positive expression rate of the three antibodies, the degree of neurological deficit, and the cerebrovascular stenosis, and the correlation of the above indicators were analyzed. The specific details are reported as follows.

## METHODS

This is a retrospective study, clinical data of 99 patients with ACI admitted to the Department of Neurology of Baoding First Central Hospital from June 2020 to December 2021 were retrospectively collected. The study was approved by the Institutional Ethics Committee of Baoding No.1 Central Hospital (No.: (2020)040; Date: May 21, 2020), and written informed consent was obtained from all participants.

### Inclusion criteria:

- Patients who met the diagnostic criteria of cerebral infarction revised by the Fourth Academic Conference of National Cerebral Vascular Disease^5^ and were confirmed by imaging examination.
- Patients with the course of the disease within two weeks.
- Patients without prior autoimmune diseases and who have not been treated with immunosuppressive agents.
- Patients aged 30-80 years.

### Exclusion criteria:

- Patients with disturbance of consciousness after cerebral infarction;
- Patients whose condition is too serious to cooperate with the study;
- Patients diagnosed with cardiopulmonary failure and other serious physical diseases;
- Patients diagnosed with severe autoimmune diseases;
- Patients with cardiac embolism.

Among all the patients, fifty-eight were males and 41 were females, aged 34-79 years, with an average age of (61.23±10.27) years; ACI course of the disease was 2-5 h, with an average course of disease (3.11±0.56) h; Body mass index (BMI) was 19-25 kg/m^2^, with an average BMI of (22.01±1.23) kg/m^2^. Education level: 20 cases of primary school or below, 32 cases of junior middle school, 38 cases of senior high school or above.

### Methods:

(1) All patients were tested for various clinical physiological indicators, such as blood routine, liver and kidney function, blood glucose, blood lipids, four items of blood coagulation, plasma D-dimer, homocysteine, electrocardiogram, head CT, head MRI, Cervical ultrasound, transcranial Doppler ultrasound; (2) Five ml fasting venous blood was drawn from all selected patients. After routine serum separation, ANA was detected by indirect immunofluorescence assay kit (EUROIMMUN, Germany) and ANCA and ACA were detected by ELISA kit (Elabscience). Reference standard for positive antibody^6^-^8^: (1) ANA positive standard: titer ≥1:100; (2) ACA positive standard: immunoglobulin IgM>1.56 U/ml, IgA>1.24 U/ml, IgG>1.42 U/ml; (3) ANCA positive standard: absorbance ratio ≥1; (3) Cerebrovascular stenosis of patients was analyzed and assessed, including anterior cerebral artery, middle cerebral artery, posterior cerebral artery, internal carotid artery, vertebral artery, basilar artery, etc., with specific evaluation indicators^9^: mild stenosis (vascular stenosis rate less than 50%), moderate stenosis (vascular stenosis rate between 50% and 69%), severe stenosis (vascular stenosis rate between 70% and 99%), occlusion (vascular stenosis rate of 100%). Vascular stenosis rate = (normal lumen diameter - stenosis site lumen diameter)/normal lumen diameter ×100%; (4) The degree of neurological deficit was assessed by the National Institute of Health Stroke Scale (NIHSS)^10^, which included 11 items (consciousness, gaze, visual field, limb ataxia, facial paralysis, upper and lower limb movement, sensation, dyslexia, language and neglect), with a total score of 0-42 points. Specific assessment criteria were as follows: mild defect (score< 4 points), moderate defect (score between 4-10 points), and severe defect (score > 10 points).

### Observation indicators:

(1) ANA, ACA and ANCA antibody test results of patients; (2) Patients’ cerebrovascular stenosis and NIHSS assessment; (3) Cerebrovascular stenosis of patients with different test results of ANA, ACA and ANCA antibodies; (4) Neurological deficits of patients with ANA, ACA and ANCA antibodies with different test results; (5) Correlation between ANA, ACA, ANCA antibodies and cerebrovascular stenosis rate and NIHSS score. The maximum follow-up time for patients in both groups was 6 months. And case data collection ceased in June 2021.

### Statistical methods:

All data in this study were analyzed and processed by the statistical software SPSS20.0. Qualitative data were expressed as rate(%), *X^2^* test was used for inter-group differences, and rank sum test was adopted for inter-group data comparison. Moreover, quantitative data were represented by (*χ̅*±*S*), and t-test was used for inter-group differences. The correlation between ANA, ACA, ANCA positive and negative antibodies as well as the rate of cerebrovascular stenosis and NIHSS score was analyzed by Pearson correlation analysis. P*<*0.05 indicates a statistically significant difference.

## RESULTS

Among 99 patients with cerebral infarction, the positive rates of ANA, ACA and ANCA antibodies were 68.69%, 70.71%, and 69.70%, respectively. See Table-I. Among 99 patients with cerebral infarction, cerebrovascular stenosis: 28 (28.28%) patients with mild stenosis, 32 (32.32%) patients with moderate stenosis, 39 (39.39%) patients with severe stenosis; NIHSS assessment: 15 (15.15%) patients had mild neurological deficits, 44 (44.44%) patients had moderate neurological deficits, and 40 (40.40%) patients had severe neurological deficits.

**Table-I:** ANA, ACA and ANCA antibody test results of patients.

Group	Number of cases	Negative (case)	Negative rate (%)	Positive (case)	Positive rate (%)
ANA antibody	99	31	31.31	68	68.69
ACA antibody	99	29	29.29	70	70.71
ANCA antibody	99	30	30.30	69	69.70

The degree of cerebrovascular stenosis between the ANA, ACA, and ANCA antibody positive group and the negative group was statistically significant by the rank sum test (*p<*0.05). See Table-II. The degree of neurological impairment between the ANA, ACA, and ANCA antibody positive group and the negative group was statistically significant by the rank sum test (*p<*0.05). See Table-III. ANA, ACA, ANCA antibody positive was moderately positively correlated with cerebrovascular stenosis rate and NIHSS score (0.40<*r*<0.60, *p<*0.05). See Table-IV.

**Table-II:** Analysis of cerebrovascular stenosis in patients with different ANA, ACA and ANCA antibody test results [cases (%)].

Group	Number of cases	Mild stenosis (28 cases)	Moderate stenosis (32 cases)	Severe stenosis (39 cases)
ANA positive group	68	15(22.06)	21(30.88)	32(47.06)
ANA negative group	31	13(41.94)	11(35.48)	7(22.58)
Z			6.353	
p			0.042	
ACA positive group	70	14(20.00)	25(35.71)	31(44.29)
ACA negative group	29	14(48.28)	7(24.14)	8(27.59)
Z			8.098	
p			0.017	
ANCA positive group	69	16(23.19)	19(27.54)	34(49.28)
ANCA negative group	30	12(40.00)	13(43.33)	5(16.67)
Z			9.348	
p			0.001	

**Table-III:** Analysis of neurological deficits in patients with different ANA, ACA and ANCA antibody test results [cases (%)].

Group	Number of cases	Mild stenosis (15 cases)	Moderate stenosis (44 cases)	Severe stenosis (40 cases)
ANA positive group	68	5(7.35)	28(41.18)	35(51.47)
ANA negative group	31	10(32.26)	16(51.61)	5(16.13)
Z		15.821		
p		0.000		
ACA positive group	70	6(8.57)	26(37.14)	37(52.86)
ACA negative group	29	9(31.03)	14(48.28)	3(10.35)
Z		17.151		
p		0.000		
ANCA positive group	69	4(5.80)	25(36.23)	39(56.52)
ANCA negative group	30	11(36.67)	19(63.33)	1(3.33)
Z		30.636		
p		0.000		

**Table-IV:** Correlation between ANA, ACA, ANCA antibodies and cerebrovascular stenosis rate and NIHSS score.

Variable	Cerebrovascular stenosis rate		NIHSS score	

	r	p	r	p
ANA positive	0.457	0.001	0.519	0.000
ACA positive	0.418	0.004	0.507	0.000
ANCA positive	0.429	0.002	0.538	0.000

## DISCUSSION

In this study, the incidence of mild, moderate and severe neurological deficits in patients with ACI was 15.15%, 44.44% and 40.40%, respectively. A specific analysis was carried out on the relationship between the degree of neurological impairment and ANA, ACA and ANCA antibodies. As the results showed, statistically significant differences were observed in the comparison of the degree of neurological impairment between the ANA, ACA and ANCA antibody positive group and the negative group, that is, the more serious the degree of neurological impairment in patients with ACI, the higher the positive rate of ANA, ACA and ANCA antibodies. It was also found in this study that the positive ANA, ACA and ANCA antibodies were moderately positively correlated with the rate of cerebrovascular stenosis and NIHSS score, further indicating that the positive expression rate of ANA, ACA and ANCA antibodies is a key influencing factor for the disease development of patients with ACI, and may participate in the abnormal pathological activities of blood vessels and nerves in the brain of patients.

It has been stated in related studies^11^ that for patients with ACI whose pathogenesis is unknown, their disease development may be closely related to the body’s autoimmune regulation. The principal manifestations include related vascular stenosis or occlusion, which in turn involves peripheral nerve cells, resulting in varying degrees of neurological deficits. ANA, ACA, ANCA antibodies may have numerous adverse effects on the structure and function of cerebrovascular endothelial cells and nerve cells in patients with ACI.

To further analyze the relationship between the above three antibodies and incidence of ACI, serum analysis of 99 patients with ACI was conducted in this study, and the positive rates of ANA, ACA and ANCA antibodies were 68.69%, 70.71% and 69.70%, respectively, indicating that the secretion levels of ANA, ACA, ANCA antibodies in patients with ACI were relatively high, which proves to a certain extent the obvious influence relationship between ANA, ACA, ANCA antibodies and the onset of ACI. It was reported in the study of Yang Lihong et al.^12^ That the high positive rate of ANA would significantly increase the risk of patients with ACI. Li Lingyu et al.^13^ and Yu Shenyi et al.^14^ Showed in their studies on ACA and ANCA antibodies that ACA and ANCA antibodies were key indicators affecting the severity and recurrence rate of ACI.

All the above studies support the findings of this study. Among 99 patients with ACI in this study, the incidence of mild, moderate and severe cerebrovascular stenosis was 28.28%, 32.32% and 39.39%, respectively. It is essential to further find out the relationship between the three antibodies and the degree of cerebrovascular stenosis, so as to analyze the explicit effects of ANA, ACA, and ANCA antibodies on the severity of vascular infarction in patients with ACI. As shown in the results of this study, statistically significant differences could be observed in the degree of cerebrovascular stenosis between the ANA, ACA and ANCA antibody positive group and the negative group, that is, the more severe the cerebrovascular stenosis, the higher the positive rate of ANA, ACA, and ANCA antibodies. This was partly consistent with the research results of Xiong Yi et al.,^15^ indicating that the severity of cerebrovascular stenosis in patients with ACI is related to the body’s autoimmune response.

ANCA may give rise to endothelial cell injury and vascular lesion in cerebrovascular and carotid arteries of patients, as well as the abnormal proliferation of intimal cells of the vessel wall. ANA and ACA antibodies generate a large number of platelets by activating the coagulation mechanism, resulting in thrombosis, further blockage of blood vessels, sharp reduction of circulation space in the blood, and in serious cases, blocked blood flow. The higher the activity of ANA, ACA, and ANCA antibodies, the more intense the degree of cerebrovascular blockage.^16^

The cerebral pia mater is an important tissue rich in brain blood vessels and nerves, in which the blood vessels and nerve are staggered winding due to its shape characteristic, causing blood circulation disorders in patients with ACI. After ischemic necrosis and softening of brain tissue, the structure and function of the peripheral nerve also showed abnormal changes, resulting in neurological defects. ANA, ACA, ANCA antibodies may not only affect the healthy state of blood vessels in the brain, but also stimulate the immune stress of brain tissue. Nerve regulation can dominate the immune function of the body, and immune cell products can also regulate neuroendocrine signals or cytokine secretion levels, affecting nerve function.^17^-^18^

With this conclusion, important indicators with clinical reference value are provided for the subsequent prevention, diagnosis and treatment of cerebrovascular stenosis and neurological deficits in patients with ACI. In addition, the test methods for ANA, ACA and ANCA antibodies are touted as safe, non-invasive and user-friendly, which are more acceptable to patients and can contribute to reducing the incidence and recurrence rate of ACI and alleviating the risk of disability.^19^

### Limitations:

There are still some shortcomings in this study. The number of subjects included in this study was limited, so the conclusions drawn may not be very convincing. In addition, this study was a retrospective study with limited data integrity and homogeneity. It is necessary to further design a randomized controlled trial to verify the conclusions of this study.

## CONCLUSIONS

To put it in a nutshell, the high positive expression rate of ANA, ACA and ANCA antibodies is a high-risk factor leading to cerebrovascular stenosis and neurological deficits in patients with ACI, providing important clinical guidance for the protection and recovery of vascular and neurological function in patients with ACI in the later period.

### Authors’ Contributions:

**HM and XX:** designed this study, prepared this manuscript, are responsible and accountable for the accuracy and integrity of the work. **CZ and JZ:** collected and analyzed clinical data. **CR and YL:** Data analysis, significantly revised this manuscript.
